# Pyrethrins Protect Pyrethrum Leaves Against Attack by Western Flower Thrips, *Frankliniella occidentalis*

**DOI:** 10.1007/s10886-012-0097-7

**Published:** 2012-03-29

**Authors:** Ting Yang, Geert Stoopen, Gerrie Wiegers, Jing Mao, Caiyun Wang, Marcel Dicke, Maarten A. Jongsma

**Affiliations:** 1 Plant Research International, Wageningen UR, P.O. Box 619, 6700 AP Wageningen, The Netherlands; 2 Laboratory of Entomology, Wageningen UR, P.O. Box 8031, 6700 EH Wageningen, The Netherlands; 3 Key Laboratory for Biology of Horticultural Plants, Ministry of Education, College of Horticulture & Forestry Sciences, Huazhong Agricultural University, Wuhan, 430070 China

**Keywords:** Pyrethrum, Pyrethrins, Western flower thrips, *Frankliniella occidentalis*, Natural pesticide, toxicity, *Tanacetum cinerariifolium*, Crop pest

## Abstract

Pyrethrins are active ingredients extracted from pyrethrum flowers (*Tanacetum cinerariifolium*), and are the most widely used botanical insecticide. However, several thrips species are commonly found on pyrethrum flowers in the field, and are the dominant insects found inside the flowers. Up to 80 % of western flower thrips (WFT, *Frankliniella occidentalis*) adults died within 3 days of initiating feeding on leaves of pyrethrum, leading us to evaluate the role of pyrethrins in the defense of pyrethrum leaves against WFT. The effects of pyrethrins on WFT survival, feeding behavior, and reproduction were measured both *in vitro* and *in planta* (infiltrated leaves). The lethal concentration value (LC50) for pyrethrins against WFT adults was 12.9 mg/ml, and pyrethrins at 0.1 % (w/v) and 1 % (w/v) had significantly negative effects on feeding, embryo development, and oviposition. About 20-70 % of WFT were killed within 2 days when they were fed chrysanthemum leaves containing 0.01-1 % pyrethrins. Chrysanthemum leaves containing 0.1 % or 1 % pyrethrins were significantly deterrent to WFT. In a no-choice assay, the reproduction of WFT was reduced significantly when the insects were fed leaves containing 0.1 % pyrethrins, and no eggs were found in leaves containing 1 % pyrethrins. Our results suggest that the natural concentrations of pyrethrins in the leaves may be responsible for the observed high mortality of WFT on pyrethrum.

## Introduction

Western flower thrips (WFT), *Frankliniella occidentalis*, is a highly polyphagous insect that causes both direct and indirect effects on plant development and health. The adults and larvae feed on epidermal and subepidermal cells of both meristematic and mature leaf and flower tissues, inhibiting plant growth and development and causing necrotic or light-reflective blotches on the tissue. Furthermore, they indirectly damage plants by transmitting tospoviruses such as tomato spotted wilt virus (Reitz, 2009). As a result, WFT has become the most serious pest in several vegetable and flower crops world-wide (Daughtrey et al., 1997; Reitz, 2009). The widespread use of chemical insecticides to control WFT has led to increasing resistance against the major classes of synthetic insecticides (Broadbent and Pree, 1997; Flanders et al., 2000; Broughton and Herron, 2009). The growing awareness and demand for insecticides that are not environmentally hazardous has stimulated the study of plant-derived compounds for pest control (Boeke et al., 2004). Such compounds could be used as natural pesticides, and in theory, genes responsible for the biosynthesis of those compounds could be isolated and transferred to crops to improve plant defense against WFT (Annadana et al., 2002; Outchkourov et al., 2004).

Among the sources of botanical pesticides, pyrethrins from pyrethrum plants (*Tanacetum cinerariifolium*) represent the economically most important class of compounds with broad usage both in homes and organic agriculture (Casida, 1973). Pyrethrins are neurotoxins that bind to voltage-gated sodium channels of neuronal cells, causing the channels to remain open (Davies et al., 2007). Pyrethrins comprise a group of six closely related esters, named pyrethrin I and II, cinerin I and II, and jasmolin I and II. They are found in all aboveground parts of the pyrethrum plants, but predominantly in the ovaries of the flower heads (Brewer, 1973). On average, the concentration of pyrethrins is about 0.1 % (dry weight) in leaves and 1-2 % (dry weight) in flowers (Baldwin et al., 1993). Assuming a water content of 90 %, pyrethrins account for around 0.01 % of the fresh weight of leaves and 0.1-0.2 % of the fresh weight of flowers. Pyrethrins are effective against a broad spectrum of insects, while their toxicity for mammals is very low, allowing their use as a preharvest spray (Casida and Quistad, 1995; Schoenig, 1995). Western flower thrips are sensitive to synthetic pyrethroids (Thalavaisundaram et al., 2008), but there is no report on the effect of natural pyrethrins against WFT. Pyrethrins might provide pyrethrum with a broad range protection against many different insect pests, but the role of pyrethrins in pyrethrum defense has not been studied.

In initial experiments, we observed that WFT adults died within one day when fed pyrethrum leaves, but that they were abundant in open flowers. Here, we tested the hypothesis that pyrethrins are responsible for protecting pyrethrum leaves against WFT by assessing adult and embryo toxicity, and by examining feeding and oviposition deterrence both *in vitro* and *in planta*.

## Methods and Materials

### Field Observation

A pyrethrum field close to Luxi, Yunnan province, China, was used for surveying thrips populations (24°27'10.34"N-103°32'21.01"E). The field was 0.5 ha in size, and the presence of insect species was monitored during the flowering period of spring 2010, when the flowers were predominantly in developmental stages 2–5 [numbered according to Casida (1973)]. To assess populations of small resident insects including thrips, flowers at each developmental stage were collected in each one of 3 blocks of the field. Each flower was taken by the stem and turned upside down into a jar containing 75 % alcohol. Flowers were fully immersed and vigorously stirred. The procedure was repeated until each jar contained the insects from 100 flowers from a single block and at a particular stage. The number of insects of each species for each stage was scored. In the case of thrips, the number of adults and larvae were scored separately. Among all collected thrips, 30 were randomly picked and identified, where possible to the species level.

### Insects and Plant Material Used in Laboratory Experiments

A population of WFT was mass-reared on flowering chrysanthemum (*Chrysanthemum morifolium* Ramat.) cv. Sunny Casa in a greenhouse under a photoperiod of L16:D8 at 25 ± 2°C. In this study, only adult female thrips were used. The chrysanthemum plants used for bioassays were from the same cultivar, but were grown in an insect-free compartment of the greenhouse under the same light and temperature conditions. All bioassays were conducted in a climate room at 20–22°C with a L16:D8 photo regime.

### Insecticide

Pyrethrum oil (70 % w/w) had been extracted from dried and ground pyrethrum flower heads with liquid CO_2_ leaving no solvent residue (Honghe Senju Biological Co. Ltd., Yunnan, China). Butylated hydroxytoluene (BHT) had been added to the oil (1 %) to prevent oxidation. We confirmed the concentration and composition of the oil by Gas chromatography–mass spectrometry comparison to a pyrethrin standard (Nguyen et al., 1998). Since the major insecticidal compounds in pyrethrum have long been known as pyrethrins (Casida, 1973), the effect of pyrethrum oil was considered to be the effect of pyrethrins. When calculating the concentrations of pyrethrins in different solutions, the percentage of pyrethrins in the oil (70 %) was taken into account. For example, 1 % (w/v) pyrethrins was prepared by dissolving 14.3 mg pyrethrum oil in 1 ml solvent.

### *In vitro* Bioassays-Toxicity Assays

The toxicity of pyrethrins was evaluated by topical application to thrips (Robb et al., 1995). Pyrethrum oil was dissolved in acetone to achieve a concentration range of 1 to 30 mg pyrethrins per ml, and the solutions were applied to the thorax with a 10-μl glass syringe at 1 μl per thrips. The droplet briefly covered the thorax of the insect and also the paper support before evaporating in a few seconds, leaving a residue both on the insect and the support. Acetone alone was used as control. After treatment, all thrips were transferred to Petri dishes containing a piece of chrysanthemum leaf embedded in an agar substrate. Mortality was assayed after 24 h by counting the number of insects that did not respond to prodding with a fine brush. Six replicates were used for each concentration, and 10 thrips were used per replicate. Percent mortality was corrected for mortality observed in acetone control using Schneider-Orelli’s formula (Schneider-Orelli, 1947). Data were analyzed using probit analysis (Finney, 1977).

### *In vitro* Bioassays-Choice Assays with Topically Applied Pyrethrins

A dual-choice leaf disk assay was used to determine the deterrent effect of pyrethrins on WFT. All leaf disks (diam 1.6 cm) were punched from chrysanthemum leaves of similar leaf age. Pyrethrum oil was dissolved in 0.2 % (v/v) aqueous Tween-80 to achieve 3 concentrations of pyrethrins: 0.01, 0.1, and 1 % (w/v). Control leaf disks were sprayed with solvent solution (0.2 % Tween-80), and test leaf disks were sprayed with the pyrethrin solutions using a Potter Precision Laboratory spray tower, which produced a uniform deposit (3 μl/cm^2^) of solution on the leaf disks. After overnight starvation, WFT were anaesthetized on ice. Groups of 10 WFT were positioned between a control and a test leaf disk placed abaxial side up and 2 cm apart on a 1.5 % (w/v) agar-bed in a Petri dish (7 cm diam). After positioning the thrips, the Petri dish was covered by a 120 μm mesh size nylon mesh lid to prevent condensation. The number of WFT on each leaf disk was recorded 0.25, 1, 2, 4, 20, and 28 h after the release of the WFT. Each concentration was replicated with 12 leaf disks. At each time point, a Student's paired *t*-test was used to assess the significance of the differences in the mean number of WFT between test and control.

### *In vitro* Bioassays-Oviposition Assays

Oviposition-deterrent effects were assayed with a non-choice method slightly modified from Annadana et al. (2002). The assay was conducted in Perspex ring cages (3 cm in length and 3.5 cm diam), which were closed with a nylon mesh at the bottom. Pollen of Scotch pine (*Pinus sylvestris* L.) was supplied in a small open tube as food source for WFT. After placing 10 WFT in a cage, the top was sealed with two layers of stretched Parafilm, with 300 μl aqueous solution in between the layers. The solutions used were water, 0.2 % Tween-80, or pyrethrins at 0.01, 0.1, or 1 % dissolved in 0.2 % Tween-80. WFT were allowed to adapt to the diet (pollen and water) for 3 d, and then every day for 5 d fresh test solution was provided. All eggs were deposited in the solutions, and were counted daily under a binocular microscope. Each solution was replicated 6 times. Data were analyzed by a one-way ANOVA and a mean separation test was conducted using LSD (α = 0.05).

### *In vitro* Bioassays-Embryo Development Assays

Around 200 WFT were kept in a Perspex ring cage (7 cm in length and 9 cm diam) to allow oviposition in a water solution as described above. Eggs laid on the same day were collected with a fine brush under a binocular microscope and then transferred to 2 layers of filter paper in Petri dishes (3.5 cm diam). The filter papers were drenched in 300 μl of assay solution (water, 0.2 % Tween-80 or pyrethrins at 0.01, 0.1, or 1 % in 0.2 % Tween-80) so that each paper was fully wetted but had no excess solution. After transferring the eggs, the Petri dishes were closed with lids and sealed with Parafilm. The developmental status of eggs was monitored every day for 6 d. To facilitate the observations, the bottoms of the Petri dishes were marked with lines that could be seen through the filter paper from the top, and the eggs were placed on filter paper along these lines. This facilitated finding the eggs under the microscope, and the viability of hatched larvae was assessed in terms of their ability to move away (>0.5 cm) from the hatch position. Four replicates of 10 eggs were used for each assay. Data were analyzed by a one-way ANOVA and mean separation test was conducted using LSD (α = 0.05).

### *In planta* Bioassays-Mortality Assays on Pyrethrum Leaves

Mature pyrethrum leaves were harvested in November from a field in the Netherlands when they were still flowering (51°59'22.08"N-5°39'44.75"E, Wageningen). Two or three pieces of leaves were placed, abaxial side up, on 1 % (w/v) agar in a Petri dish (7 cm diam). After transferring 10 WFT to each Petri dish, dishes were covered with lids with gauze. Petri dishes with two leaf disks (1.6 cm diam) of chrysanthemum leaves, with a total mass similar to the mass of the pyrethrum leaf samples, or with only agar were used as controls. Six replicates were carried out for each treatment. The mortality of WFT was recorded daily for 3 d.

### *In planta* Bioassays-Choice Assays

To test the *in planta* activity of pyrethrins against WFT, pyrethrins were infiltrated into whole chrysanthemum leaves as described by Ratcliff et al. (2001). Leaf disks (diam 1.6 cm) were punched from the infiltrated leaves, avoiding the infiltration points so that WFT would not contact pyrethrins directly except at the edge of the disk. In the initial experiments, we infiltrated only water into the leaves and determined that on average 29.1 mg (± 2.1 mg) water could be infiltrated into each leaf disk (6 replicates). As the fresh weight of each leaf disk was on average 45.3 mg (± 1.2 mg), we infiltrated 0.025, 0.25, or 2.5 % pyrethrins solution to bring the concentrations to 0.01, 0.1, or 1 % pyrethrins. Leaf disks infiltrated with 0.2 % Tween-80 were used as control. The assay and data analysis were conducted as described above for the choice assays with topically applied pyrethrins. The number of WFT on each leaf disk was recorded 0.25, 1, 2, 4, 20, and 28 h after the release of the WFT.

### *In planta* Bioassays-Reproduction Assays

To test the effects of pyrethrins on oviposition and hatching of larvae, WFT were assayed with chrysanthemum leaf disks as described by De Kogel et al. (1997), with slight modifications. Leaf disks were punched from untreated leaves, from leaves infiltrated with 0.2 % Tween-80, or from leaves containing 0.01, 0.1, or 1 % pyrethrins in Tween solution. WFT were placed on leaf disks (1.2 cm diam, 2 WFT/disk), which were embedded, abaxial side up, on agar in wells of 24-well Greiner plates. Plates were covered with Parafilm, and every well was carefully sealed by pressing the Parafilm on the edge of each well. WFT were allowed to oviposit for 48 h and were then removed, with simultaneous assessment of mortality. Subsequently, half of the leaf disks from each plate were used to determine the number of eggs, and the other half of the leaf disks were used to determine the number of hatched larvae. To determine the number of eggs, the leaf disks were boiled in water for 3 min so that the eggs were clearly visible under a binocular microscope with transmitting light. To determine the number of hatched larvae, the leaf disks were transferred to Petri dishes containing water and incubated in a climate chamber (25°C, L16:D8) for 5 d to allow the larvae to hatch. The hatched larvae were counted under a binocular microscope. One plate containing 24 identical leaf disks was used for each treatment. Data were analyzed by a one-way ANOVA and mean separation test was conducted using LSD (α = 0.05).

## Results

### Natural Distribution of Insects in Pyrethrum Fields

**Fig. 1:**
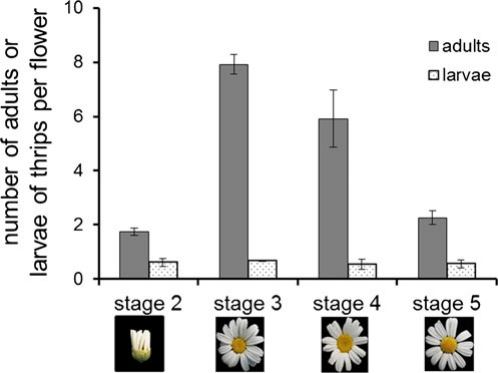
Distribution of thrips adults and larvae across different developemental stages of pyrethrum flowers in the field. Error bars indicate SE (*N* = 300 per stage). Stage 2, vertical ray florets; stage 3, horizontal ray florets and first row of disk florets open; stage 4, 3 rows of disk florets open; stage 5, all disk florets open

Our field survey in China showed that several thrips species were the most abundant (98 %) insects on pyrethrum flowers (Table 1). In addition, a few *Nysius* species (Heteroptera: Lygaeidae) (1.9 %) and lacewing larvae (Neuroptera) (0.05 %) were found. A total of 30 individuals were identified to species level; the thrips species found were mainly *Thrips tabaci* (44 %), *Frankliniella occidentalis* (western flower thrips, or WFT, 25 %), and *Thrips flavus* (22 %). The number of thrips in flowers was dependent on the flower’s developmental stage (Fig. 1). The number of thrips increased until stage 3 (the first row of disk florets are open), and then decreased in later stages. The thrips found inside flowers were mainly adults. Larvae accounted for 7-26 % of the total number of thrips per flower, depending on the flower developmental stage (Fig. 1).

**Table 1:** Frequencies of small insect species living on pyrethrum flowers in the field

Insects^a^	Frequency (%)	Species^b^	Frequency (%)
Thripidae (thrips)	98	*Thrips tabaci*	43
		*Frankliniella occidentalis*	25
		*Thrips flavus*	21
		*Thrips palmi*	3
		Other species	6
*Nysius* sp.	1.9	n.d.	1.9
*Chrysoperla/Chrysopa* sp. (lacewing larva)	0.05	n.d.	0.05

^a^A total of 1200 insects were collected to count the frequencies of different insects. ^b^ A total of 30 thrips were used to identify species. N.d., not determined

### Effect of Pyrethrum Leaves on Mortality of WFT

We assayed the suitability of pyrethrum leaves as a food substrate for WFT. Mortality could be as high as 80 % within 3 days, although the degree of mortality depended on the plant source (data not shown). When only water and agar were provided, with no plant-based food, only 20-30 % WFT died in 3 days. All WFT feeding on control chrysanthemum leaves remained alive during the 3-day-experiment. This showed that the mortality of WFT was caused by a toxic principle of pyrethrum leaves rather than deterrence or starvation.

The toxic principle of pyrethrum plants against insects has long been known to be a group of 6 pyrethrin esters (Casida, 1973). We were, therefore, interested in specifically testing the effect of pyrethrins against WFT.

### *In vitro* Insecticidal and Deterrent Effects

To determine the effects of pyrethrins against WFT, pyrethrins were tested *in vitro* at different concentrations on adult mortality, feeding, oviposition, and embryo development.

The mortality of WFT female adults increased with the concentration of topically applied pyrethrins in the range of 1 to 30 mg/ml. Probit analysis showed that the LC_50_ and LC_90_ of pyrethrins was 12.9 mg/ml (with 95 % confidence limit of 10.9-14.8 mg/ml) and 39.0 mg/ml (with 95 % confidence limit from 30.7 to 50.4 mg/ml), respectively.

**Fig. 2:**
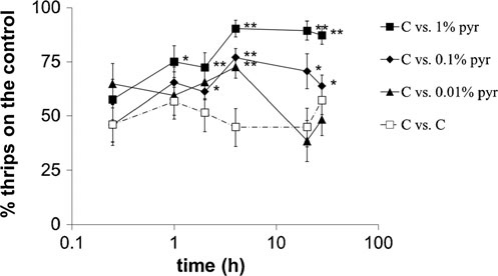
Dual choice assays of western flower thrips on chrysanthemum leaf disks sprayed with 0.2 % Tween (control) or 0.2 % Tween with 0.01 %, 0.1 % or 1 % pyrethrins. The presence on either leaf disk was visually recorded 0.25, 1, 2, 4, 20 and 28 h after WFT release. The x-axis represents ^10^log-transformed time data. Asterisks indicate significant differences to the control (*: *P* < 0.05; **: *P* < 0.01). C, control. Pyr, pyrethrins. Error bars indicate SE (*N* = 120 per treatment)

Thrips were significantly deterred from feeding by 0.1 % and 1 % pyrethrins (Fig. 2). When given a choice between chrysanthemum leaf disks coated with 0.2 % Tween (control) or 0.1 % added pyrethrins, after 2 h significantly more (61-77 % of thrips) settled on control leaf disks. Pyrethrins at 1 % were more highly deterrent. Within 1 h, 72-90 % of thrips chose control leaf disks. For both concentrations of pyrethrins, the maximum deterrent effect was reached at 4 h. Application of 0.01 % pyrethrins on leaf disks did not show significant deterrent effects except at the 4 h time point (Fig. 2).

**Fig. 3:**
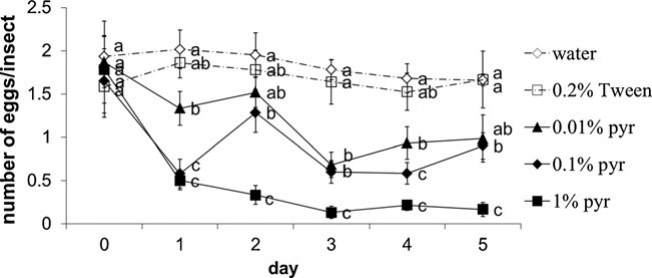
The number of eggs deposited by western flower thrips when supplied with different concentrations of pyrethrins starting on Day 1. Data points with the same letter within days are not significantly different, *P* < 0.05. Pyr, pyrethrins. Error bars indicate SE (*N* = 60 per treatment)

Pyrethrins negatively affected oviposition by WFT (Fig. 3). The carrier, 0.2 % Tween-80, did not affect the oviposition of thrips compared to water throughout the experiment, but WFT oviposited significantly fewer eggs with increasing pyrethrin concentrations during the 5-day experiment (Fig. 3).

**Fig. 4:**
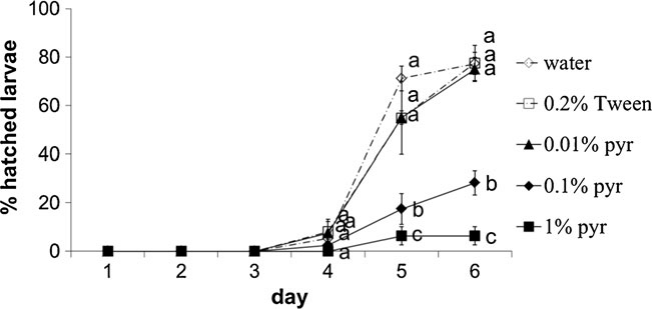
Percentage of larvae hatching from western flower thrips eggs during incubation with different concentrations of pyrethrins. Data points with the same letter within days are not significantly different, *P* < 0.05. Pyr, pyrethrins. Error bars indicate SE (*N* = 40 per treatment)

**Fig. 5:**
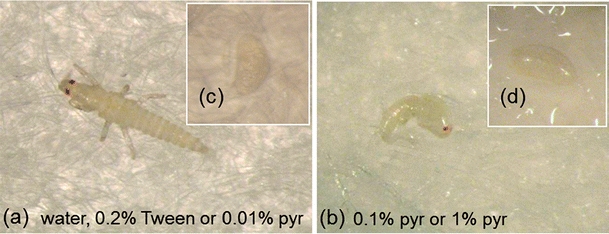
Effects of pyrethrins on embryo development of western flower thrips at day 5. (a), larva hatched in solutions of water, 0.2 % Tween or 0.01 % pyrethrins at day 5; (b), abnormally developed embryos in solutions of 0.1 % and 1 % pyrethrins at day 5; (c) and (d), embryos before treatment

The development of eggs was inhibited by 0.1 % and 1 % pyrethrins. About 80 % of larvae hatched when the eggs were incubated with water, 0.2 % Tween, or 0.01 % pyrethrins, while only 28 % or 6 % of the larvae hatched when the eggs were incubated with 0.1 % or 1 % pyrethrins, respectively (Fig. 4). In the latter two treatments, the embryos that did not develop into larvae had severely stunted and abnormal shapes (Fig. 5), and dried out after a few days.

### *In planta* Insecticidal and Deterrent Effects

To study *in planta* activity of pyrethrins against WFT, thrips were assayed with chrysanthemum leaves that had been infiltrated with pyrethrins to contain 0.01, 0.1, or 1 % pyrethrins. In this experiment, the pyrethrins could not be contacted directly by thrips except by feeding, and the source of nutrition consisted of leaves instead of pollen.

In the reproduction assay, thrips fed with chrysanthemum leaf disks containing pyrethrins exhibited higher mortality and lower reproduction rates compared to those fed with untreated leaf disks or leaf disks containing 0.2 % Tween (Table 2).

**Table 2:** Mortality, number of eggs and hatched western flower thrips larvae per leaf disk on chrysanthemum leaf disks infiltrated with different concentrations of pyrethrins

Treatment of leaf disks	Mortality (%)	Eggs	Hatched larvae
Untreated leaf disks	0 a	2.0 ± 0.4 a	1.4 ± 0.3 a
Leaf disks containing 0.2 % Tween	0 a	1.7 ± 0.5 a	1.3 ± 0.3 ab
Leaf disks containing 0.01 % pyrethrins	25.0 ± 6.7 b	1.3 ± 0.3 ab	0.8 ± 0.2 b
Leaf disks containing 0.1 % pyrethrins	29.2 ± 7.9 b	0.7 ± 0.2 bc	0.1 ± 0.1 c
Leaf disks containing 1 % pyrethrins	68.8 ± 9.3 c	0 c	0 c

Values (mean ± SE, *N* = 48 per treatment) followed by the same letter within a column are not significantly different (ANOVA: *P* > 0.05)

**Fig. 6:**
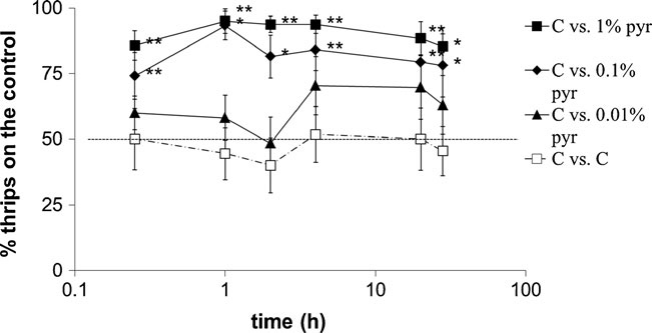
Percentage of western flower thrips settled on the control chrysanthemum leaf disk in dual choice assays of leaf disks containing 0.2 % Tween with or without 0.01 %, 0.1 %, or 1 % pyrethrins. The solutions were infiltrated into chrysanthemum leaves. The choices were recorded 0.25, 1, 2, 4, 20, and 28 h after WFT release. The x-axis represents ^10^log-transformed time data. Asterisks indicate significant differences to the control (*: *P* < 0.05; **: *P* < 0.01). C, control. Pyr, pyrethrins. Error bars indicate SE (*N* = 120 per treatment)

In the dual-choice assay, chrysanthemum leaves containing 0.1 % and 1 % pyrethrins showed significant deterrent effects on thrips within 15 min of release (Fig. 6). A total of 74-93 % of the thrips settled on the control leaf disk when the other leaf disk contained 0.1 % pyrethrins, and 85-95 % thrips settled on the control leaf disk when the other leaf disk contained 1 % pyrethrins. Chrysanthemum leaves containing 0.01 % pyrethrins did not show significant deterrent effects.

## Discussion

Pyrethrins, well-known natural insecticidal compounds, are found exclusively in and extracted from the composite flowers of pyrethrum (*Tanacetum cinerariifolium*), which belongs to Anthemideae tribe within the Astaraceae family (Casida and Quistad, 1995). Remarkably, the potential role of pyrethrins in pyrethrum plant defense has not been studied. Here, we report that western flower thrips (WFT) adults thrive on pyrethrum flowers, but die within a few days on pyrethrum leaves. The hypothesis that pyrethrins are responsible for protecting pyrethrum leaves against WFT was tested by spraying or infiltrating pyrethrins to leaves of chrysanthemum, a related pyrethrins-free species belonging to the same tribe. We assessed toxicity to the adult and embryo stages of WFT, and negative effects on feeding and oviposition both *in vitro* and *in planta,* and found that the natural concentrations of pyrethrins present in leaves have strong negative effects on WFT. We speculate that the thrips found on pyrethrum flowers survive on pollen that is devoid of pyrethrins (T. Yang, unpublished data).

For many populations of WFT, resistance has been reported for some synthetic insecticides (Espinosa et al., 2005). Furthermore, many synthetic insecticides are harmful for human health and the environment. It is relevant, therefore, to find natural insecticides effective against WFT. Previously, several other plant-derived compounds were tested for their insecticidal effects against WFT adults. For example, carvacrol at 1 % and thymol at 0.1 % and 1 % significantly reduced the oviposition rate of WFT when these compounds were sprayed on leaf disks, but neither compound affected the feeding activity of WFT (Sedy and Kosehier, 2003). Salicylaldehyde (0.1 % and 1 %) and methyl salicylate (0.1 % and 1 %) were also tested. Within 24 h of applying 1 % methyl salicylate to bean or cucumber, the feeding and oviposition activities of thrips females were significantly reduced (Koschier et al., 2007). The effect on the insect could be a result of changes in the plant induced by methyl salicylate, since it is a plant hormone involved in induced resistance (Pieterse et al., 2009). A series of commercially available plant-derived essential oils tested at recommended concentrations (0.02-0.5 %), including neem oil, rosemary oil, peppermint oil, garlic oil, and cottonseed oil, caused less than 30 % mortality within 7 days (Cloyd et al., 2009). Compared to other plant-derived compounds, pyrethrins are highly effective against WFT. Our results showed that 0.1 % and 1 % pyrethrin solutions sprayed on leaf disks significantly deterred WFT at 4 h, and topically applied pyrethrins were toxic to adults at an LC_50_ value of 12.9 mg/ml (1.3 %). By mimicking the natural site of pyrethrin accumulation by infiltration of leaves, we found that 1 % pyrethrins caused 69 % mortality and completely inhibited oviposition. Furthermore, 0.1 % pyrethrins was strongly deterrent and resulted in abortion of 95 % of the embryos, while as little as 0.01 % pyrethrins caused 25 % mortality in 2 days. We propose, therefore, that the natural concentrations of pyrethrin in pyrethrum leaves, around 0.01 % by fresh weight, accounts for the observed high mortality of thrips adults on this plant.

Insecticides have not been reported previously to affect the development of WFT embryos. WFT eggs are embedded in plant tissues (Childers, 1997), and as a result they are unlikely to be affected by non-systemic chemicals that are applied on the surface of plants. However, pyrethrins naturally accumulate inside pyrethrum tissues, stored in what appear to be unstructured intercellular cavities (M.A. Jongsma, unpublished observations). Therefore, besides feeding and oviposition deterrence, the embryo-toxic effect of pyrethrins is a third component that contributes to their effect for plant defense against WFT (Figs. 4 and 5).

Compared to some synthetic insecticides, the toxicity of natural pyrethrins against WFT in the absence of synergists was not high. In previous studies using topical application methods, the LC_50_ values of insecticides tested against susceptible WFT strains ranged from 10 to 83 μg/ml for pyrethroids, 20 to 960 μg/ml for carbamates, and 49 to 522 μg/ml for organophosphates (Espinosa et al., 2005; Robb et al., 1995). The LC_50_ value of pyrethrins against WFT by topical application was determined as 12.9 mg/ml, and the action of pyrethrins was, therefore, 10 to 1000-fold weaker than for these synthetic pesticides. On the other hand, pyrethrins did show much stronger negative effects on feeding behavior and reproduction, which may be explained by the action of pyrethrins on the nervous system, resulting in disordered function of excitable (nerve and muscle) cells (Bradberry et al., 2005). At 0.01 % (100 μg/ml), pyrethrins not only caused mortality of adults and embryos, but also significantly reduced oviposition (Table 2). All these factors together cumulatively affect the life history parameters. As a result WFT damage on pyrethrin-containing leaves may be virtually absent, and virus transmission also may be strongly reduced. We hypothesize that if plants such as closely related chrysanthemum species, which do not contain any pyrethrins, were genetically engineered to produce pyrethrins, their resistance to WFT in leaves could be significantly improved.
